# Wnt/Chemerin Signaling Involved in Exercise Training Preventing Diaphragm Dysfunction Induced by Cigarette Smoke

**DOI:** 10.3390/biomedicines14061382

**Published:** 2026-06-18

**Authors:** Peijun Li, Jian Li, Yingqi Wang, Xiaoyu Han, Yuanyuan Cao, Meiling Jiang, Yidie Bao, Weibing Wu, Xiaodan Liu

**Affiliations:** 1 School of Rehabilitation Science, Shanghai University of Traditional Chinese Medicine, Shanghai 201203, China; lpj0227@163.com (P.L.); lijianfitness@126.com (J.L.); wangyingqii@163.com (Y.W.); baoyidie@126.com (Y.B.); 2 Department of Sports Rehabilitation, Shanghai University of Sport, Shanghai 200438, China; hanxy78@163.com (X.H.); caoyuanyuan97@163.com (Y.C.); jml_1994@163.com (M.J.); 3 Institute of Rehabilitation Medicine, Shanghai Academy of Traditional Chinese Medicine, Shanghai 201203, China; 4 Engineering Research Center of Traditional Chinese Medicine Intelligent Rehabilitation, Ministry of Education, Shanghai 201203, China

**Keywords:** exercise training, diaphragm dysfunction, cigarette smoke, inflammation, chemerin/CMKLR1, Wnt/β-catenin

## Abstract

**Objectives**: The current study examined whether exercise training alleviates cigarette smoke (CS)-induced diaphragm dysfunction by modulating inflammation through the Wnt and Chemerin signaling pathways. **Methods**: Mechanical stretching was applied for 3 consecutive days to explore the effects on cell proliferation and chemerin/chemokine-like receptor 1 (CMKLR1) expression in C2C12 cells pretreated with lipopolysaccharide. Male wild-type (WT) and CMKLR1 knockout (KO) mice (6–8 weeks old) were exposed to CS for 6 months (1–2 h a day, 6 days a week) to determine the role of chemerin/CMKLR1 in the progression of diaphragm dysfunction. Given that Wnt/β-catenin is a potential modulator of chemerin/CMKLR1, its expression was detected in CS-exposed mice and mice subjected to treadmill exercise training after CS exposure. Wnt/β-catenin agonist lithium chloride (LiCl) and antagonist XAV939 were then intraperitoneally injected into the CS-exposed mice during exercise training to further investigate their potential synergistic effects with exercise training on improving CS-induced diaphragm dysfunction. Isolated diaphragm contraction strength and fiber cross-sectional area were measured to determine the diaphragm dysfunction. **Results**: Mechanical stretching improved the proliferation level of myoblasts and decreased inflammation and CMKLR1 protein expression (*p* < 0.05). The KO mice showed diminished diaphragm dysfunction compared with the WT mice after long-term CS exposure. Combined LiCl and exercise training further enhanced the improvement of diaphragmatic isolated strength in mice exposed to CS (*p* < 0.01), activated the protein degradation and synthesis pathways, and decreased IL-1β level (*p* < 0.05). Combined XAV939 and exercise training significantly decreased chemerin protein level (*p* < 0.01). **Conclusions**: Exercise training can downregulate inflammation levels and improve diaphragm dysfunction in CS-exposed mice, partially by enhancing Wnt expression and reducing abnormally activated chemerin.

## 1. Introduction

Cigarette smoke (CS) is one of the most potent risk factors for premature mortality and morbidity worldwide [1]. Long-term CS has been well-documented to cause chronic obstructive pulmonary disease (COPD) and other respiratory diseases, including lung cancer. In the early stages of CS, the diaphragm experiences an increased influx of inflammatory cells, which might contribute to muscular atrophy and oxidative capacity loss [2,3]. CS, which contains more than 7000 chemicals, includes components that directly impair diaphragmatic contractility and disrupt mitochondrial morphology and function in the diaphragm, leading to a decline in its contraction function [4]. Lipopolysaccharide (LPS), a constituent of the outer wall of Gram-negative bacteria and a contaminant of CS, inhibits myogenic differentiation via a Toll-like receptor (TLR) 4-NF-κB-dependent pathway, thereby promoting muscle atrophy in C2C12 cells [5]. CS directly and indirectly impairs muscle function, and slow-twitch muscles are particularly vulnerable to CS and exhibit significant oxidative-to-glycolytic fiber conversions [6]. As the primary respiratory muscle, the diaphragm is directly subjected to chest wall remodeling, an increased load and other pathologies [7]. Consequently, its susceptibility to CS has attracted growing research attention. When the diaphragm is affected, it contributes to dyspnea, respiratory failure, and even premature death in COPD [8,9].

The clinical diagnosis of diaphragm dysfunction is based on static and dynamic imaging tests [10]; in animals, its diagnosis is considered comprehensive and includes morphology and contraction performance [11]. Following 24 weeks of CS exposure, the diaphragm exhibits a sustained reduction in mass and fiber diameter, accompanied by functional decompensation and changes in gene expression [12,13]. Systemic inflammation has been intensively explored as a potential mechanism for CS-induced diaphragm dysfunction; increased levels of inflammatory factors interleukin (IL)-1β, IL-6, and tumor necrosis factor (TNF)-α in lung tissues may overflow into extrapulmonary tissues through blood circulation, thereby activating the ubiquitin–proteasome pathway, inducing apoptosis or programmed cell death, and impairing muscle regeneration capacity [14]. Chemerin, also known as tazarotene-induced gene 2 protein or retinoic acid receptor responder protein 2, is associated with elevated inflammatory factors and metabolic syndrome [15]. Among its known receptors, chemokine-like receptor 1 (CMKLR1) is recognized as the primary receptor mediating its inflammatory responses in human-derived muscle cells [16]; CMKLR1 modulates muscle regeneration and contractility [17]. Overall, chemerin/CMKLR1 signaling plays a critical role in excessive inflammation-induced muscle dysfunction. Early evidence indicates that the transcription factor-peroxisome proliferator-activated receptor gamma (PPARγ) directly regulates chemerin expression through a putative PPARγ response element sequence within the chemerin promoter [18]. The T-cell factor/Lymphoid enhancer-binding factor domain in β-catenin can directly interact with and regulate the expression of PPARγ [19]. Our previous study on the mechanisms of aerobic exercise training in improving COPD pulmonary function revealed that its benefits were linked to the modulation of inflammation via the Wnt/β-catenin/PPARγ pathway [20,21]. This suggests that Wnt/β-catenin may act upstream of chemerin/CMKLR1 in COPD pulmonary rehabilitation [22]. However, direct evidence for Wnt/β-catenin regulating chemerin/CMKLR1 signaling in skeletal muscle remains limited, let alone evidence for the role of Wnt/Chemerin signaling in the alleviation of CS-induced diaphragm dysfunction by exercise training. Therefore, we hypothesized that exercise-induced mechanical loading suppresses excessive inflammation via chemerin/CMKLR1 signaling in the CS-exposed diaphragm, with Wnt/β-catenin potentially acting as an upstream regulator. This proposed Wnt/Chemerin axis constitutes a druggable pathway to counteract CS-induced respiratory muscle failure.

In vitro cell experiments in C2C12 myoblasts and in vivo animal experiments in C57BL/6 mice were conducted. The present study first investigated whether mechanical stretching affects chemerin/CMKLR1 expression in a C2C12 inflammation model; CMKLR1 knockout mice were selected to circumvent the systemic metabolic disturbances associated with chemerin knockout mice [23], thereby allowing us to directly explore the specific role of chemerin/CMKLR1 in CS-induced diaphragm dysfunction in mice; the study further examined the effects of exercise training combined with Wnt/β-catenin modulation and finally clarified the involvement of Wnt/Chemerin in the prevention of CS-induced diaphragm dysfunction by exercise training.

## 2. Materials and Methods

### 2.1. Reagents and Antibodies

Reagents used included Dulbecco’s modified Eagle medium (DMEM) (Gibco, Grand Island, NY, USA), fetal bovine serum (Gibco, Grand Island, NY, USA), 100 U/mL penicillin and 100 μg/mL streptomycin (Gibco, Grand Island, NY, USA), lipopolysaccharide (LPS) (Sigma, St. Louis, MO, USA), Lithium chloride (LiCl) (Sigma, St. Louis, MO, USA), 3,5,7,8-Tetrahydro-2-[4-(trifluoromethyl)phenyl]-4H-thiopyrano[4,3-d]pyrimidin-4-one (XAV939) (MCE, Monmouth Junction, NJ, USA), Cell Counting Kit-8 (CCK8) (Dojindo Laboratories, Kumamoto, Japan), a mouse IL-1β ELISA kit (Wellbio, Shanghai, China), a mouse TNF-α ELISA kit (Wellbio, Shanghai, China), RIPA buffer (Wellbio, Shanghai, China), a Bicinchoninic Acid Assay kit (Wellbio, Shanghai, China), protein loading buffer (5×, Wellbio, Shanghai, China), TRIzol (Invitrogen, Carlsbad, CA, USA), a First Strand cDNA Synthesis Kit (Invitrogen, Carlsbad, CA, USA), and a QuantityNova SYBR Green PCR Kit (Qiagen, Hilden, Germany).

The antibodies used included FBX32 rabbit pAb (YN3108), TRI63 polyclonal antibody (YN0691), ChemR23 polyclonal antibody (YT0897), Wnt-1 polyclonal antibody (YT 4907), and β-catenin polyclonal antibody (YT5866), all purchased from Immunoway (Plano, TX, USA). The chemerin polyclonal antibody (10216-1-AP) and MyoD polyclonal antibody (18943-1-AP) were purchased from Proteintech (Rosemont, IL, USA). GAPDH (WB0197) was purchased from Wellbio (Shanghai, China).

### 2.2. Cells and Animals

The murine C2C12 myoblast cell line was utilized as a well-established and tractable in vitro model to investigate the interactive effects of inflammatory and mechanical stimuli on myogenic cells. C2C12 myoblasts were purchased from the National Collection of Authenticated Cell Cultures (Shanghai, China) and cultured in DMEM with 10% fetal bovine serum and 1% penicillin-streptomycin at 37 °C and 5% CO_2_ in a humidified CO_2_ incubator. The growth medium was changed every other day. When the cells reached 80% confluence, they were passaged at a split ratio of 1:2 in the growth medium and then seeded into each well of a 6-well plates. Low passages at P3 to P8 cells were used for cell experiments.

Six-to-eight-week-old male wild-type (C57BL/6J) mice (Zhejiang Vitalriver Laboratory Animal Company, Jiaxing, Zhejiang, China) and CMKLR1-/- (C57BL/6J) mice (Shanghai Model Organisms, Shanghai, China) were purchased and maintained in a specific pathogen-free environment in the Experimental Animal Center, Shanghai University of Traditional Chinese Medicine, China. All experimental procedures were ethically approved by the Shanghai University of Traditional Chinese Medicine Animal Care Committee (No. PZSHUTCM210312012) and conformed to ARRIVE guidelines. All mice used in this study were age-matched and male.

### 2.3. Experimental Groups

To investigate the effect of mechanical stretching on the C2C12 inflammation model, LPS was applied as a canonical inducer for inflammation to simulate the inflammatory component of the complex pathological mechanisms induced by CS. C2C12 myoblasts were randomly divided into a control group (no treatment), an LPS group (LPS treatment), and an LPS and mechanical stretching group (LPS treatment followed by mechanical stretch). All experiments were conducted three times independently.

For the study of the role of chemerin/CMKLR1 in CS-induced diaphragm dysfunction, 12 WT mice and 12 CMKLR1-/- mice were randomly assigned into a control group (exposed to air) and a model group (exposed to CS) at a 1:1 ratio.

Eighteen WT C57BL/6 mice were randomly allocated to the control group (exposed to air), the model group (exposed to CS), and the exercise training group (exposed to CS followed by exercise training). After the impact of exercise training on Wnt/β-catenin was observed, 36 WT C57BL/6 mice were assigned to the control group (exposed to air), the model group (exposed to CS), the LiCl group (exposed to CS followed by LiCl treatment), the LiCl + exercise training group (exposed to CS followed by LiCl treatment and exercise training), the XAV939 group (exposed to CS followed by XAV939 treatment), and the XAV939 + exercise training group (exposed to CS followed by XAV939 treatment and exercise training) to clarify the role of Wnt/Chemerin in diaphragm dysfunction improvement by exercise training.

The randomization was performed using a computer-generated random number sequence by an independent researcher who was not involved in the subsequent experimental procedures or outcome assessments.

### 2.4. Protocol of the Model

C2C12 myoblasts were treated with 0, 0.1, 0.2, 0.5, 1, 2 and 5 mg/mL LPS for 24 h [24]. In brief, 100 μL of cells (5 × 10^4^/mL) were seeded into 96-well plates and cultured for 24 h, followed by the addition of 10 μL of LPS at different concentrations and another culture for 24 h. Cell proliferation levels were then detected using CCK8 to select an appropriate LPS concentration for subsequent experiments.

In brief, mice were exposed to CS using a whole-body exposure system (PAB-S200, Beijing Bestlab High-Tech Co., Ltd., Beijing, China) by placing them in a chamber (80 × 60 × 58 cm^3^). The smoke generated by commercially filtered cigarettes (10 mg of tar, 0.9 mg of nicotine, 12 mg of CO per cigarette) was delivered into the chamber. Temperature, O_2_, CO_2_, and CO levels in the chamber were measured continuously during the exposure period. In accordance with our previous study [17], the amount of CS received by mice increased gradually with smoking time. In this study, a CS protocol of 1 h a day in the 1st week and 1 h twice a day from the 2nd to the 25th week, 6 days a week, was used. The results of the gas sensor showed that the O_2_ concentration was maintained between 18% and 20%, ensuring the mice were not subjected to hypoxia during the CS period. The CO concentration was maintained at 310–440 ppm for weeks 1–7, 410–540 ppm for weeks 8–13, and 510–640 ppm for weeks 14–25. A laser dust particle counter (NOHAWK NK800, Tianjin Liaowang Optoelectronics Technology Co., Ltd., Tianjin, China) was used to measure the particulate matter (PM) concentrations inside the chamber immediately after cigarette combustion ceased. The results showed that during weeks 1–7, PM10 ranged from 9487 to 9961 μg/m^3^ and PM2.5 ranged from 9241 to 9785 μg/m^3^; during weeks 8–13, PM10 ranged from 10,744 to 12,112 μg/m^3^ and PM2.5 ranged from 9987 to 10,688 μg/m^3^; during weeks 14–25, PM10 ranged from 14,688 to 15,211 μg/m^3^ and PM2.5 ranged from 13,412 to 14,345 μg/m^3^. Age-matched and room air-exposed mice served as controls.

### 2.5. Protocol of Exercise Training

C2C12 myoblasts pretreated with LPS were seeded into 6-well plates (BioFlex, FlexCell International Corporation, Burlington, PA, USA). Before mechanical stretching, the cells were cultured for 24 h and then stretched at 15% elongation and 0.5 Hz frequency for 2, 4, and 6 h for 3 consecutive days by using a computer-controlled stretching instrument (Flexcell FX-6000 TM Tension System, FlexCell International Corporation, Burlington, PA, USA).

After CS exposure, the mice underwent exercise training on a motorized treadmill during which the inclination was maintained at 0° (flat). Initially, the mice underwent an exercise adaptation period (5 m/min, 10–60 min/session, 6 days), followed by a treadmill exercise test to evaluate their maximal exercise capacity, which corresponded to the maximal velocity reached in the test [25]. The test was performed with a 5 min warm-up (5 m/min), followed by an increase in treadmill speed (2 m/min every 3 min) until exhaustion. Aerobic exercise training began on week 2 of the protocol and was performed at 55% of the maximal velocity reached in the test (60 min/session, 1 session/day, 6 days/week, 8 weeks) [26].

### 2.6. Protocol of Medicine Administration

For the regulation of Wnt/β-catenin, the agonist LiCl and antagonist XAV939 were used. The doses of modulators of Wnt were adopted from previous studies [27,28]. LiCl (200 mg/kg body weight) and XAV939 (2.5 mg/kg body weight) were dissolved in 100 μL of sterile water and then intraperitoneally injected into mice 30 min before exercise training, once per day at 6 days/week for 8 weeks.

### 2.7. Indicator Measurement

**Figure 1:**
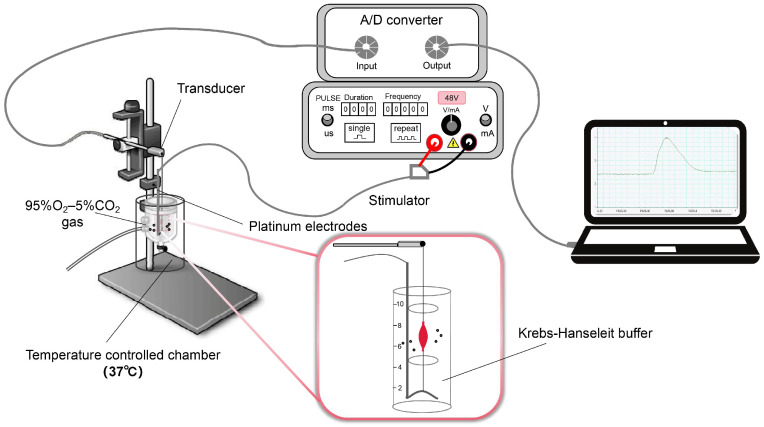
Schematic diagram of diaphragm function test. A 3 mm wide diaphragm strip was bathed in Krebs-Hanseleit buffer (pH 7.4, 37 °C) continuously gassed with O_2_ (95%) and CO_2_ (5%). A suture was used to fix one end of the strip to the bottom of the bath and the other end to the tension sensor. The two loop electrodes were positioned on either side of the diaphragm strip. After the strip was placed in the bath, the stimulator was applied to stimulate the diaphragm strip at 40 V with 500 μs electrical field stimulation. The diaphragm contraction was recorded by a transducer, and the data were converted and recorded in LabChart Software (V8.1.13). The diaphragm contraction strength (g) was used for later analysis.

**Diaphragm function test:** Figure 1 shows a diagram of the test. Following thoracotomy, a 3 mm wide diaphragm strip was cut from the central tendon toward the rib along the direction of the diaphragm fibers. The isolated strip was bathed in Krebs-Hanseleit buffer (118.0 mmol/L NaCl, 4.7 mmol/L KCl, 1.2 mmol/L MgSO_4_·7H_2_O, 1.2 mmol/L KH_2_PO_4_, 25.0 mmol/L NaHCO_3_, 2.5 mmol/L CaCl_2_, 11.0 mmol/L glucose, pH 7.4, 37 °C) continuously gassed with O_2_ (95%) and CO_2_ (5%). An automatic organ bath (Panlab Harvard Apparatus, Holliston, MA, USA) and PowerLab 4/35 data acquisition hardware (ADInstruments, Oxford, UK) were used to maintain the physiological environment of the isolated strips and record the diaphragm tension, respectively. A suture was used to fix one end of the strip to the bottom of the bath and the other end to the tension sensor. The two loop electrodes were positioned on either side of the diaphragm strip. A four-channel stimulator (LE12406, Panlab Harvard Apparatus, Holliston, MA, USA) was applied to stimulate the diaphragm strip at 40 V with 500 μs electrical field stimulation. The diaphragm contraction strength was recorded for later analysis.

**Diaphragm histology:** The diaphragms of mice were removed and fixed in 4% paraformaldehyde solution. After paraffin embedding, 4 to 6 μm thick sections of diaphragm tissues were stained with hematoxylin and eosin (HE). The HE sections were then imaged using a brightfield microscope. The cross-sectional area (CSA) of diaphragm fibers was measured to evaluate morphological changes. Five randomly selected different representative nonoverlapping fields at 200× magnification were selected from each lung section. Assessment of pathological slides and subsequent quantification were performed in a blinded manner. Specifically, all slides and corresponding digital images were randomly coded, and the analysts were kept unaware of the group identity until after all measurements were completed.

**Western blot:** Diaphragm tissue was harvested, and protein lysates were prepared using RIPA buffer containing protease and phosphatase inhibitors. Then, they were homogenized, sonicated for 2–3 min with 10 to 15 s pulses on ice, and centrifuged at 12,000 rpm at 4 °C for 20 min. Protein concentration was determined using a BCA kit. Protein loading buffer (5×) was added to the lysis solution and denatured for 10 min. Proteins were separated by 12% SDS-PAGE gel, transferred to a polyvinylidene fluoride (PVDF) membrane, and the PVDF membrane was blocked with 5% skim milk for 2 h at room temperature and incubated with the primary antibodies against Atrogin-1, 1:1000; MuRF-1, 1:1000; MyoD, 1:3000; wnt1, 1:2000; β-catenin, 1:1000; chemerin, 1:1000; CMKLR1, 1:1000 at 4 °C overnight. After washing the membrane in TBST for 5 min/3 times, the membrane was incubated with the corresponding secondary antibodies (GAPDH, 1:2000) for 2 h at room temperature. The protein bands in the membranes were then visualized using enhanced electrochemiluminescence detection reagents. Western blot signals were acquired using an imager (Tanon Biotechnology, Shanghai, China) and subsequently processed and analyzed via ImageJ software (1.54g). The level of the target protein was normalized to GAPDH, expressed as the fold change relative to the control group, and then subjected to statistical analysis for intergroup comparisons. To ensure objectivity, all blot images were assigned random codes, and the investigator performing the densitometry was blinded to the sample groups during the entire measurement process.

**qPCR:** Total RNA was extracted from the cells using TRIzol. For quantitative polymerase chain reaction (qPCR), RNA was reverse transcribed to cDNA using a First Strand cDNA Synthesis Kit in accordance with the manufacturer’s instructions. Real-time PCR was performed with 45 cycles using the QuantityNova SYBR Green PCR Kit. The amplification step was completed by the Roche LightCycler^®^ 480 II Real-Time PCR System (Roche, Basel, Switzerland). Gene expression was normalized to that of GAPDH, and the data were analyzed using the 2^−ΔΔCt^ method. The primer sequences are listed in Table 1.

**ELISA:** After stretching, the culture medium was immediately replaced with a fresh one and incubated for 24 h. The culture medium was then collected and centrifuged at 12,000 rpm for 5 min. The abdominal cavity of the mice was opened, and 0.5–0.7 mL of blood was collected from the inferior vena cava, kept at room temperature for 30 min, and centrifuged at 12,000 rpm and 5 °C for 5 min. Analysis was performed in accordance with the kit instructions.

**CCK8:** A CCK8 assay was used to detect cell proliferation. After stretching, the culture medium was replaced with a fresh one and incubated for 24 h. Afterward, 10% of the culture medium volume with CCK8 solution was added to the medium, followed by further incubation for 2 h. Optical density (OD) was detected at 450 nm using a microplate reader (Biotek, Winooski, VT, USA).

### 2.8. Statistical Analysis

Statistical analysis was performed using GraphPad Prism 7.0 software (San Diego, CA, USA). All data are presented as the mean and standard deviation (SD). The Shapiro–Wilk test was used to verify data normality, and Levene’s homogeneity of variance test was applied to verify data homogeneity of variance. Differences were evaluated with one-way analysis of variance, two-way analysis of variance or nonparametric tests as appropriate. Differences were considered significant at *p* < 0.05.

## 3. Results

### 3.1. Mechanical Stretching Decreased Chemerin/CMKLR1 Expression in the C2C12 Inflammation Model

**Figure 2:**
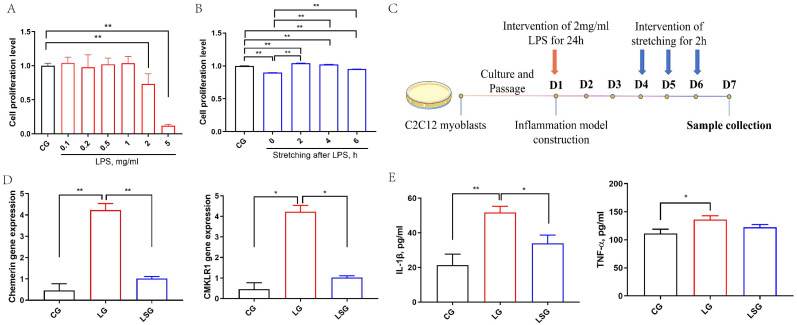
Mechanical stretching decreases the chemerin/CMKLR1 expression in the C2C12 inflammation model. (**A**). The proliferation level of C2C12 myoblasts after LPS treatment at different concentrations, which was measured by CCK8; (**B**). the proliferation level of LPS-pretreated C2C12 myoblasts after mechanical stretching, which was measured by CCK8; (**C**). schematic diagram of cell experimental timeline: C2C12 myoblasts were treated with 2 mg/mL of LPS for 24 h and then seeded into 6-well plates, cultured for 24 h, and finally stretched at 15% elongation and 0.5 Hz frequency for 2 h for 3 consecutive days; (**D**). the relative mRNA expression of chemerin and CMKLR1, which were determined by qPCR; (**E**). relative levels of IL-1β and TNF-α in cell suspension, which were measured by ELISA. Values are presented as means ± SD; differences were evaluated with one-way analysis of variance. * *p* < 0.05; ** *p* < 0.01; CCK8, Cell Counting Kit-8; CG, control group; CMKLR1, chemokine-like receptor 1; D, day; IL, interleukin; LG, LPS group; LPS, lipopolysaccharide; LSG, LPS+ mechanical stretching group; qPCR, quantitative polymerase chain reaction; SD, standard deviation; TNF, tumor necrosis factor. Cell experiments were conducted three times independently.

To determine the impact of the inflammatory environment on muscle atrophy, we used a mature C2C12 myoblast cell line, a good model for muscle stem cells that can display fast proliferation, differentiation, and contractile myotube formation ability. CCK8 results showed that 2 and 5 mg/mL of LPS significantly reduced the proliferation level of C2C12 myoblasts (*p* = 0.0006 and *p* < 0.0001, Figure 2A), and 5 mg/mL of LPS seriously affected cell survival. This finding suggests that 2 mg/mL of LPS can reduce cell proliferation levels without affecting cell survival and thus can be used to prepare C2C12 myoblast inflammation models.

To determine the effect of mechanical stretching on C2C12 cell proliferation under inflammatory conditions, we applied cyclic stretching for 2, 4, and 6 h every day over 3 consecutive days. The CCK8 results showed that mechanical stretching significantly improved the proliferation of LPS-pretreated C2C12 myoblasts (*p* < 0.0001 compared with the control), but the effect gradually decreased with time (Figure 2B).

Chemerin is a chemokine secreted by white adipose tissue and can play a pro- or anti-inflammatory regulatory role in disease-related inflammatory responses [15]. The results showed that the mRNA expression of chemerin and CMKLR1 was significantly increased in the C2C12 inflammation model, whereas mechanical stretching significantly reduced their levels (*p* < 0.05, Figure 2C,D). Accordingly, the levels of the inflammatory cytokines IL-1β and TNF-α were significantly increased in the supernatant of the C2C12 inflammation model (*p* = 0.0007 and *p* = 0.011), and mechanical stretching reduced the elevated levels of inflammatory cytokines to some extent (*p* = 0.0042 for IL-1β, Figure 2C,E). These data suggest that chemerin/CMKLR1 expression exhibits a significant response to inflammation and exercise training at the cellular level.

### 3.2. CMKLR1 Knockout Alleviates Diaphragm Dysfunction in CS-Exposed Mice

**Figure 3:**
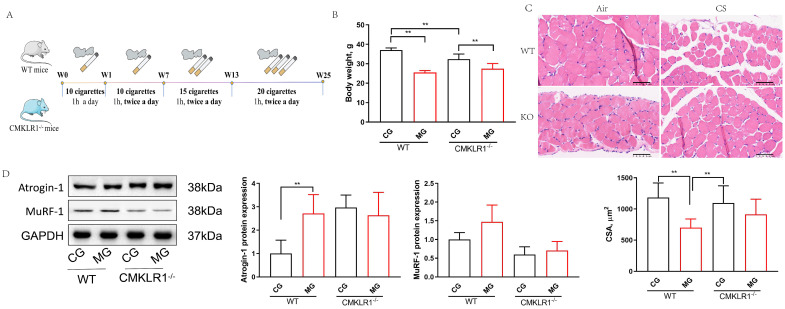
CMKLR1 knockout alleviates diaphragm dysfunction in CS-exposed mice. (**A**). Schematic diagram of CS exposure: 10 cigarettes 1 h a day in the 1st week, 10 cigarettes 1 h twice a day from the 2nd to 7th weeks, 15 cigarettes 1 h twice a day from the 8th to 13th weeks, and 20 cigarettes 1 h twice a day from the 14th to 25th weeks, 6 days a week; (**B**). body weight (*n* = 6 each group); (**C**). HE-staining images of the diaphragm (bar = 50 μm) and the CSA (*n* = 3 per group); (**D**). WB bands and the relative protein expression in Atrogin-1 and MuRF-1 (*n* = 6 per group). Values are presented as means ± SD; differences were evaluated with two-way analysis of variance. ** *p* < 0.01; CS, cigarette smoking; CSA, cross-sectional area; CG, control group; HE, hematoxylin and eosin; KO, knockout; MG, model group; SD, standard deviation; W, week; WT, wild-type.

To clarify the role of chemerin/CMKLR1 in CS-induced diaphragm dysfunction, we subjected WT mice and CMKLR1-/- mice to long-term, incremental, whole-body CS exposure. After 25 weeks of CS exposure, the body weight of both mouse groups significantly decreased (*p* < 0.0001 for both, Figure 3A,B), and the degree of weight loss in the CMKLR1-/- mice was less than in the WT mice. Quantitative analysis of diaphragm HE staining showed a decrease in CSA after CS, especially in the WT mice (*p* < 0.01, Figure 3A,C). In addition, the protein expression of Atrogin-1 in the diaphragm was significantly increased only in the WT mice (*p* = 0.0001, Figure 3A,D). Accordingly, the CMKLR1-/- mice showed slow growth and development in weight, and the CS-exposed CMKLR1-/- mice showed less prominent features of diaphragm dysfunction.

### 3.3. Wnt/β-Catenin Is Partly Involved in Exercise Training Regulating Chemerin/CMKLR1 to Improve CS-Induced Diaphragm Dysfunction

**Figure 4:**
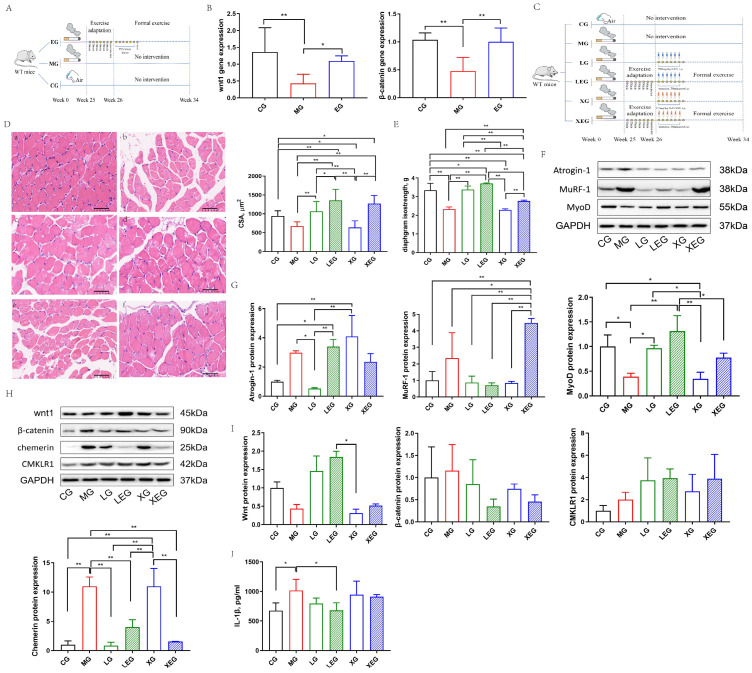
The role of Wnt in exercise training improving diaphragm dysfunction induced by CS. (**A**). Schematic diagram of the exercise training experiment: WT mice were divided into CG, MG, and EG. MG and EG mice were exposed to 25 weeks of CS, and after CS exposure, EG mice were submitted to an exercise protocol, which included 1 week of exercise adaptation and an 8-week formal exercise period; (**B**). the relative mRNA expression of Wnt1 and β-catenin, which were determined by qPCR (*n* = 6 each group); (**C**). schematic diagram of the exercise training and medicine administration experiment: WT mice were divided into CG, MG, LG, LEG, XG and XEG. MG, LG, LEG, XG and XEG mice were exposed to 25 weeks of CS; then, LG and LEG mice were submitted to LiCl intraperitoneal injection and XG and XEG mice were submitted to XAV939 intraperitoneal injection. For LEG and XEG mice, the intraperitoneal injection was conducted 30 min before exercise training; (**D**). HE-staining images of the diaphragm (bar = 50 μm) and the CSA (a, CG; b, MG; c, LG; d, LEG; e, XG; f, XEG; *n* = 3 per group); (**E**). force generation assessment of diaphragms isolated from mice (*n* = 6 per group); (**F**,**G**). WB bands and the relative protein expression in Atrogin-1, MuRF-1 and MyoD (*n* = 3 each group); (**H**,**I**). WB bands and the relative protein expression in Wnt1, β-catenin, chemerin and CMKLR1 (*n* = 3 each group); (**J**). the levels of IL-1β in serum, which were measured by ELISA (*n* = 4 each group). Values are presented as means ± SD; differences were evaluated with one-way analysis of variance. * *p* < 0.05; ** *p* < 0.01; CG, control group; CMKLR1, chemokine-like receptor 1; CS, cigarette smoking; CSA, cross-sectional area; EG, exercise training group; HE, hematoxylin and eosin; IL, interleukin; LEG, LiCl + exercise training group; LG, LiCl group; LiCl, Lithium chloride; MG, model group; qPCR, quantitative polymerase chain reaction; SD, standard deviation; WT, wild-type; XAV939, 3,5,7,8-Tetrahydro-2-[4-(trifluoromethyl)phenyl]-4H-thiopyrano[4,3-d]pyrimidin-4-on; XEG, XAV939 + exercise training group; XG, XAV939 group.

Previously, we reviewed the possible role of chemerin/CMKLR1 in COPD pulmonary rehabilitation and speculated that Wnt/β-catenin may be the upstream modulator [22]. In this study, we found that exercise training impacts chemerin/CMKLR1 expression in the C2C12 inflammation model, and CMKLR1 plays a critical role in CS-induced diaphragm dysfunction in mice. Furthermore, exercise training increased the Wnt1 and β-catenin expression levels in the CS-exposed mice diaphragms (Figure 4A,B). Wnt/β-catenin modulation with or without exercise training was conducted in CS-exposed mice with diaphragm dysfunction to clarify the role of Wnt/β-catenin in exercise training-induced improvements in diaphragm dysfunction.

Quantitative analysis of histological results showed that LiCl or XAV939 combined with exercise training significantly improved the CSA of diaphragm fibers in mice exposed to CS (*p* < 0.01, Figure 4C,D). Diaphragm function was evaluated through the force generation assessment of the diaphragm isolated from mice. The results showed that LiCl improved the diaphragm strength of the mice exposed to CS, and this improvement was further enhanced after LiCl was combined with exercise training (*p* < 0.0001 compared with MG, Figure 4E). XAV939 decreased the diaphragm strength of the mice exposed to CS to some extent, and its combination with exercise training reversed the weakness (*p* = 0.001). Accordingly, assessment of protein levels in the diaphragm (Figure 4C,F,G) showed that LiCl significantly decreased Atrogin-1 in the MG (*p* = 0.0106), whereas its combination with exercise training increased Atrogin-1 expression (*p* = 0.0028). XAV939 combined with exercise training increased MuRF-1 expression, attributed to the further activation of protein degradation and synthesis pathways after its combination with exercise training.

In addition, LiCl significantly decreased the chemerin protein level in MG (*p* < 0.0001). XAV939 increased the chemerin protein level in MG, compared with LiCl with or without exercise training (*p* < 0.0001 and *p* = 0.002). XAV939 combined with exercise training significantly decreased the chemerin protein level, compared with XAV939 (*p* = 0.0001). The protein level of CMKLR1 was not completely consistent with that of chemerin; LiCl and XAV939 combined with or without exercise training increased these expression levels compared to MG (*p* > 0.05). The protein expression of Wnt was decreased in CS-exposed mice diaphragms; LiCl combined with or without exercise training obviously increased the Wnt protein level, XAV939 slightly inhibited the Wnt protein level and XAV939 combined with exercise training reversed the effects. A significant difference in Wnt protein level was observed between XAV939 and LiCl combined with exercise training (*p* < 0.05, Figure 4C,H,I). The protein level of β-catenin in MG was not completely consistent with the gene level and was not completely consistent with that of Wnt. However, no significant difference was observed among groups (*p* > 0.05). Analysis of inflammation in the circulatory system showed that LiCl combined with exercise training significantly decreased the level of IL-1β (*p* < 0.05, Figure 4C,J).

## 4. Discussion

The present study demonstrated that myoblast inflammation with impaired proliferation and overexpression of chemerin/CMKLR1 can be alleviated by mechanical stretching. In mice with CS exposure, CMKLR1 played a critical role in diaphragm atrophy. Wnt agonists can enhance the inhibition of exercise training on the chemerin, whereas Wnt antagonists have the opposite effect, while both of them regulate systemic inflammation and diaphragm dysfunction. In summary, exercise training improves CS-induced diaphragm dysfunction, which involves downregulation of the chemerin/CMKLR1 axis and concurrent activation of the Wnt/β-catenin pathway.

Muscle dysfunction is closely related to impaired satellite cell proliferation. As activated satellite cells, C2C12 myoblasts can self-proliferate and are a good tool for studying muscle tissue pathology. The effects of LPS on skeletal muscle are primarily mediated by the TLR4-NF-κB inflammatory pathway, whereas those of CS involve more complex mechanisms including autophagy, pyroptosis and inflammation [29,30]. As one of the means to simulate exercise training in vivo, mechanical stretching can exert different effects by adjusting its parameters, including amplitude, time, and frequency. Some reports showed that mechanical stretching could promote myoblast proliferation [30,31,32,33], which is consistent with the present study, although the effect gradually decreased with the prolongation of single stretching time. The importance of chemerin/CMKLR1 in the proliferation and differentiation of myoblasts has been confirmed. Recombinant chemerin significantly reduces MyoD, myogenin, and MyHC levels and increases ROS levels in C2C12 myoblasts [34]. Chemerin expression may increase during myoblast differentiation through autocrine/paracrine pathways, promoting C2C12 cell proliferation and inhibiting differentiation [35]. Therefore, the present study used LPS to construct a sustained, low-level inflammatory cell model in which the levels of the pro-inflammatory cytokines IL-1β, TNF-α, and CMKLR1 mRNA were significantly increased, but decreased after mechanical stretching. This finding indicates that chemerin/CMKLR1 exhibits a distinct response to inflammation and exercise training at the cellular level.

CS causes pathological changes in the diaphragm, which occur earlier than the pathological changes in the lung tissue of C57BL/6 mice [36]. As the exposure time of CS increases, both the structure and function of the diaphragm are damaged [12,13]. Considering that chemerin/CMKLR1 responds to mechanical stretching at the cellular level, which may be related to improved proliferation and reduced inflammation, we further determined the role of chemerin/CMKLR1 in CS-induced diaphragm dysfunction. Given the current lack of widely available, well-characterized systemic chemerin knockout mouse models, this study employed a strategy targeting the disruption of its key receptor, CMKLR1, to specifically block the downstream signaling transduction mediated by the chemerin/CMKLR1 axis. No significant difference in growth and survival was observed after ablation of chemerin/CMKLR1 signaling [37]. However, delayed development, significantly reduced body weight and somite myotome components, and subtle muscle deficiency were observed in the mice with ablation of chemerin/CMKLR1 signaling [38]. The mice with ablation of chemerin/CMKLR1 signaling also exhibited reduced clinical features compared with the WT mice after subacute (4 weeks) or chronic (24 weeks) CS exposure [39]. In the present study, the body weight of the CMKLR1 KO mice was significantly lower than that of the WT mice, and the CS-induced degradation of diaphragm proteins and structural damage were more severe in the WT mice compared with those in the CMKLR1 KO mice. Taken together, these results suggest that chemerin/CMKLR1 plays a critical role in diaphragm dysfunction, which is consistent with a previous study showing that increased chemerin expression and biological activity are often unique manifestations of inflamed tissues and local areas [40]. Ablation of chemerin/CMKLR1 signaling can restore mitochondrial dysfunction caused by chemerin, supporting the important role of chemerin/CMKLR1 in regulating mitochondrial remodeling and muscle function [41]. Therefore, we speculate that chemerin/CMKLR1 regulates inflammation and participates in the progression of diaphragm dysfunction, making it a potential target for exercise training.

**Figure 5:**
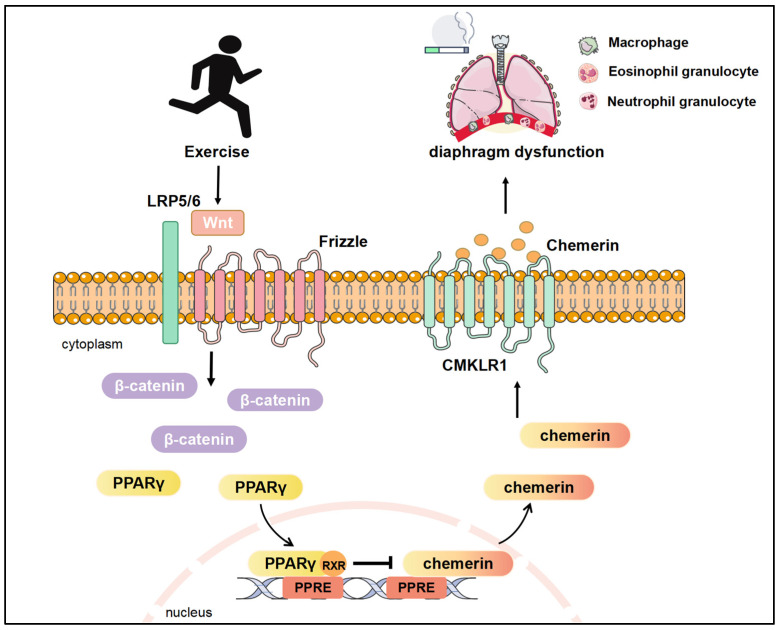
The involvement of Wnt/Chemerin in exercise training improving CS-induced diaphragm dysfunction. Exercise training may enhance Wnt expression, which could potentially lead to upregulation of PPARγ. Given that PPARγ directly represses chemerin gene transcription via a PPRE sequence in its promoter, this would provide a mechanistic link to the observed downregulation of chemerin. Reduced chemerin bioavailability is, in turn, associated with decreased CMKLR1 activation and attenuated inflammation. This cascade of molecular events constitutes a plausible model that aligns with the concurrent improvement in CS-induced diaphragm dysfunction following exercise training. CMKLR1, chemokine-like receptor 1; LRP5/6, lipoprotein receptor-related protein 5/6; PPARγ, peroxisome proliferator-activated receptor gamma; PPRE, putative PPARγ response element; RXR, retinoid X receptor.

The Wnt/β-catenin signaling pathway is one of the key factors in muscle repair. Our previous research speculated that the Wnt/β-catenin pathway is an upstream pathway in chemerin/CMKLR1 regulation by exercise training [22]. Therefore, we conducted an exploratory study and found that LiCl or XAV939 combined with exercise training could reduce the increased chemerin protein expression in the diaphragms of the CS-exposed mice. Analysis of systemic inflammation showed that LiCl combined with exercise training reduced IL-1β levels in the CS-exposed mice. In addition, LiCl improved diaphragm strength and CSA, and this effect was further enhanced when combined with exercise training. The damaging effect of XAV939 on diaphragm CSA in the CS-exposed mice was alleviated after combined exercise training. LiCl, a widely used glycogen synthase kinase 3 (GSK3) inhibitor, induces phosphorylation of GSK3α (ser21) and GSK3β (ser9) to suppress its kinase activity. This leads to β-catenin stabilization and accumulation while also modulating additional signaling pathways such as NF-κB and autophagy [42,43]. In the present study, LiCl treatment significantly increased Wnt protein expression; however, the change in β-catenin did not fully parallel that of Wnt. This discrepancy may be attributed to the pleiotropic effects of LiCl, which can also stimulate the β-catenin-independent pathway (e.g., GSK3β-independent and metabolic effects) [44]. In addition, chemerin overexpression inhibits Wnt/β-catenin pathway activity by promoting β-catenin phosphorylation and degradation [45]. CMKLR1 expression can be regulated by Wnt through β-catenin-mediated transactivation at the proximal Wnt response elements sequence [46]. Interestingly, while chemerin overexpression promotes inflammation paracrinically, it does not directly affect CMKLR1 expression [47]. In our experiments, CMKLR1 protein levels remained unchanged following LiCl or XAV939 treatment, suggesting that Wnt signaling may influence this axis primarily via chemerin expression rather than its receptor. This could be mediated by the direct regulation of chemerin by PPARγ [18], a transcription factor that often functions antagonistically to Wnt signaling. Many downstream effectors of Wnt sustain and amplify inflammation, and these complex, often antagonistic interactions with PPARγ may reinforce each other, creating a vicious cycle [19]. Notably, PPARγ activation may also increase β-catenin transcription and nuclear translocation, potentially enhancing Wnt target gene expression [48]. Taken together, the relationship between Wnt/β-catenin and chemerin/CMKLR1 is not linear or unidirectional but constitutes a dynamic network characterized by bidirectional crosstalk. Considering the responsiveness of the membrane-associated signaling molecules Wnt and chemerin to exercise training, we present a working model that may explain how exercise training improves CS-induced diaphragm dysfunction (Figure 5). In this model, exercise-induced upregulation of Wnt signaling is associated with increased PPARγ expression which, in turn, correlates with the downregulation of chemerin and attenuation of the associated inflammatory response. The involvement of Wnt/Chemerin provides a new conceptual framework for developing clinical interventions against COPD-related diaphragm dysfunction. Targeting it—for instance, by inhibiting chemerin activity or activating Wnt and PPARγ transcription—represents a potential therapeutic strategy to mitigate diaphragm injury driven by excessive inflammation. However, the complexity of Wnt signaling cascades poses significant challenges for drug development and clinical application [49]. Agonists of PPARγ (like rosiglitazone and pioglitazone) are already approved for type 2 diabetes, and the structural flexibility and diverse ligand-binding modes of PPARγ offer a molecular basis for developing drugs with differing efficacy and safety profiles [50]. In addition, exercise training-induced alterations in plasma composition are increasingly recognized as a potential treatment strategy. Preclinical studies show that systemic factors, such as extracellular vesicles and anti-inflammatory exerkines released into the circulation after exercise, can mimic the protective effects of exercise, preserving skeletal muscle health and dampening inflammation [51,52].

### Limitations

This study has some limitations. First, in vitro cell experiments using LPS as an inducer do not recapitulate several key mechanisms of CS and may differ from in vivo animal models using CS as an inducer. Future studies utilizing CS extract or specific reactive carbonyls present in CS would be valuable to extend these findings to a more disease-relevant context. Second, the use of C2C12 myoblasts rather than diaphragm satellite cells or differentiated diaphragm-derived myotubes may not fully capture diaphragm-specific biology. Future studies employing stretched, differentiated diaphragm-derived myotubes would be valuable to confirm our findings. Third, the control mice received only routine cage handling and were not subjected to the daily treadmill acclimation and placement procedures, which made it difficult to entirely rule out the contribution of differential non-specific stress responses between groups. Future studies incorporating a sham-running control (e.g., daily placement on a stationary or very slow-moving treadmill) would help to further dissect exercise-specific effects from procedural stressors. Fourth, this study only detected the total level of chemerin in the diaphragm. Further detection of chemerin isoforms with actual biological activity is needed in the future. Fifth, the knockout of CMKLR1 is not diaphragm-specific, and the loss of CMKLR1 in other organs might have a confounding effect. Sixth, the observed inconsistencies in the protein expression levels of Wnt/β-catenin and chemerin/CMKLR1 warrant further investigation. Additionally, assessing PPARγ expression in future studies would help to further elucidate the specific mechanism involved. Seventh, an a priori sample size calculation was not performed before the animal experiments. To address this limitation, we performed a post hoc power analysis using G*power 3.1 software with a two-tailed t-test, which yielded a statistical power of 0.99 (α = 0.05, Cohen’s d = 3.6 derived from the actual experimental data). This value exceeds the conventional threshold of 0.8, indicating that the sample size was adequate to detect the observed group differences in the primary outcomes. Nevertheless, formal prospective sample size calculations will be conducted in subsequent studies to strengthen the robustness and generalizability of the findings.

## 5. Conclusions

Chemerin/CMKLR1 regulates inflammation and plays a critical role in the progression of CS-induced diaphragm dysfunction. Wnt is partly involved in the regulation of exercise training by chemerin to improve CS-induced diaphragm dysfunction. Future studies using gain- or loss-of-function approaches for specific pathway components are required to formally test this mechanistic interaction and establish causality. Further confirmation is needed to identify the specific chemerin isoform. Future studies in female mice and older animals are needed to assess the generalizability of the findings.

## Figures and Tables

**Table 1:** Sequences of primer.

Genes		Primer
Rarres2	Forward	5′-CGGAGTGCACAATCAAACCA-3′
	Reverse	5′-TCAGAATTGGGCAGTGGACT-3′
Cmklr1	Forward	5′-TTTCTTCTTGAGCTCCCCGT-3′
	Reverse	5′-GGCCAAGCTGAAGTTGTTGA-3′
Wnt1	Forward	5′-GCTGCAGTGACAACATCGAT-3′
	Reverse	5′-CTTGGCGCATCTCAGAGAAC-3′
Ctnnb1	Forward	5′-CCCTGAGACGCTAGATGAGG-3′
	Reverse	5′-TGTCAGCTCAGGAATTGCAC-3′
GAPDH	Forward	5′-AGGTCGGTGTGAACGGATTTG-3′
	Reverse	5′-TGTAGACCATGTAGTTGAGGTCA-3′

## Data Availability

The raw data supporting the conclusions of this article will be made available by the authors on request.

## Abbreviations

The following abbreviations are used in this manuscript:

CCK8	Cell Counting Kit-8
CMKLR1	Chemokine-like receptor 1
CS	Cigarette smoke
CSA	Cross-sectional area
COPD	Chronic obstructive pulmonary disease
DMEM	Dulbecco’s modified Eagle medium
GSK3	Glycogen synthase kinase 3
IL	Interleukin
KO	Knockout
LiCl	Lithium chloride
LPS	Lipopolysaccharide
LRP5/6	Lipoprotein receptor-related protein 5/6
HE	Hematoxylin and eosin
qPCR	Quantitative polymerase chain reaction
OD	Optical density
PM	Particulate matter
PPARγ	Peroxisome proliferator-activated receptor gamma
PPRE	Putative PPARγ response element
PVDF	Polyvinlidene fluoride
ROS	Reactive oxygen species
RXR	Retinoid X receptor
SD	Standard deviation
TLR	Toll-like receptors
TNF	Tumor necrosis factor
WT	Wild-type
XAV939	3,5,7,8-Tetrahydro-2-[4-(trifluoromethyl)phenyl]-4H-thiopyrano[4,3-d]pyrimidin-4-one
